# Lipase Stability in Structured Lipid Synthesis: The Interplay of Substrate Characteristics and Strategies to Improve Its Operational Performance—A Critical Review

**DOI:** 10.1111/1750-3841.71175

**Published:** 2026-06-24

**Authors:** Evelyn Ling Lee, Eng‐Seng Chan, Lee Fong Siow, Cher Pin Song, Yee‐Ying Lee

**Affiliations:** ^1^ School of Science Monash University Malaysia, Jalan Lagoon Selatan, Bandar Sunway Subang Jaya Selangor Malaysia; ^2^ Department of Chemical Engineering, School of Engineering Monash University Malaysia Jalan Lagoon Selatan, Bandar Sunway Subang Jaya Selangor Malaysia; ^3^ MIPO Biorefinery Lab, School of Engineering Monash University Malaysia, Jalan Lagoon Selatan, Bandar Sunway, Subang Jaya Selangor Malaysia

**Keywords:** biocatalysts’ operational stability, esterification, interesterification, lipase‐active biocatalysts, lipid substrates’ characteristics, structured lipids synthesis

## Abstract

Structured lipids (SLs) have attracted growing industrial interest due to their associated health benefits. Lipases, as highly selective and versatile biocatalysts, are central to the sustainable synthesis of SLs attributed to their ability to operate under mild reaction conditions. However, the limited operational stability of lipases remains a major bottleneck, directly affecting process efficiency, product yield, and economic feasibility. While considerable attention has been devoted to enzyme engineering and process optimization, the influence of lipid substrate characteristics on lipase stability has received comparatively little scrutiny. This review critically examines feedstock properties, such as oxidative status, free fatty acid composition, and water activity, that affect lipase performance and deactivation during SL synthesis, where unoptimized substrate characteristics have been shown to reduce lipase stability by two‐ to sevenfold. It further discusses mitigation strategies, including substrate pretreatment with protein, adsorbents, and antioxidant, as well as process design enhancements, which have demonstrated up to sevenfold extensions in operational stability. This review highlights the importance of integrating substrate quality management into biocatalytic process design to enhance lipase operational stability and reusability. By elucidating the interplay between substrate properties and enzyme stability, this work provides a foundation to promote the valorization of nontraditional lipid sources, such as agro‐industry by‐products and underutilized oils, within circular bioeconomy frameworks, thereby supporting the development of more flexible, robust, cost‐effective, and scalable biocatalytic processes for SL production.

## 1 Introduction

As market demand for functional foods accelerate, structured lipids (SLs) have gained recognition as vital health‐promoting lipids with growing applications in food, nutraceutical, and medical sectors. SLs are modified triacylglycerols (TAGs) designed to deliver specific nutritional and functional benefits through modification of fatty acid composition and positional distribution on the glycerol backbone relative to their native forms (Hong et al. 2023). The specific positional distribution of fatty acids on the glycerol backbone is generally determined by using nuclear magnetic resonance spectroscopy, thin layer chromatography coupled with and gas chromatography or liquid chromatography‐ mass spectrometry techniques (Zhang et al. 2020). Examples include medium‐ and long‐chain triacylglycerols (MLCTs), human milk fat substitutes (HMFS), and diacylglycerol (DAG), which are increasingly incorporated into formulations for their performance advantages.

Typically, the enzymatic synthesis of SLs, primarily using lipases offers a more selective and environmentally friendly alternative to conventional chemical methods, fulfilling the criteria for sustainable bioprocessing. The reusability of lipases makes them ideal for application across different industries such as food, pharmaceuticals, cosmetics, and detergents (Ali et al. 2023; Remonatto et al. 2022) which is evidenced by the robust growth of the global lipase market, projected to expand at a compound annual growth rate (CAGR) of 6.3%–12.5% through 2030 (Fortunate Business Insight 2024; Stratistics MRC 2024).

Despite these advantages, the economic feasibility for SLs synthesis poses a significant challenge, constrained by the high cost of enzymes and lipid feedstock. For instance, lipid substrates accounted for 78% and enzymes for 21% of the total process cost in the enzymatic production of α‐monolaurin (Mustafa et al. 2023). To address this, researchers are exploring cost‐reduction strategies such as the utilization of nontraditional lipid sources, including underutilized oils and agro‐industrial by‐products, which is also aligns with principles of sustainability and the circular bioeconomy. Simultaneously, enzyme immobilization is an essential strategy, as it enhances enzyme reusability. However, even with immobilization, lipase inactivation remains a significant barrier to process scalability due lipase vulnerability to thermal, mechanical, or chemical environmental stress.

Current knowledge indicates that previous studies on biosynthesis efficiency have predominantly adopted an enzyme‐centric perspective, focusing on protein engineering and advanced immobilization to promote lipase stability (Albuquerque et al. 2016; Chandra et al. 2025; Coelho and Orlandelli 2021; Matsumoto et al. 2019; Ozdemir Babavatan et al. 2020; Song et al. 2025). While these approaches have yielded significant improvements, they often implicitly treat enzyme deactivation as an intrinsic and unavoidable limitation. Furthermore, the role of the reaction system, specifically, the characteristics of the lipid feedstock has been largely overlooked or discussed only in a fragmented manner. It should be noted that the composition and degree of refinement of lipid substrates introduce variables that can profoundly impact enzyme stability. For example, the presence of oxidation products, water activity, and the level and acyl chain length of free fatty acids (FFAs) can act as potent stressors, accelerating enzyme deactivation. This challenge is further amplified by the growing industrial interest in incorporating highly unsaturated lipids, such as docosahexaenoic acid (DHA) and eicosapentaenoic acid (EPA) into SLs synthesis. The extreme susceptibility of these polyunsaturated fatty acids (PUFAs) to oxidation can generate a cascade of reactive species that affect the product quality and the biocatalyst performance. This represents a critical gap, as substrate can directly modulate lipase conformation and catalytic efficiency, ultimately governing deactivation kinetics. A comprehensive and systematic evaluation of how substrate attributes influence lipase operational stability has received disproportionately little attention in both academic research and industrial process development.

This review critically evaluates the lipid substrate characteristics on the operational stability of lipases for the synthesis of SLs. We provide a consolidated and comprehensive collection of these factors, summarize and propose practical mitigation strategies, and identifies current industrial challenges for potential future research directions. By addressing this underexplored interface between substrate quality and enzyme performance, this review aims to complement existing enzyme engineering approaches to support the development of more flexible, robust, cost‐effective, and scalable biocatalytic processes for SLs production.

## 2 Methodology

This review was conducted following the principles of the preferred reporting items for systematic reviews and meta‐analyses (PRISMA) 2020 statement to ensure transparency, reproducibility, and rigor in the literature selection process. Given the critical and qualitative nature of this review, a systematic approach to literature identification and screening was adopted, though formal meta‐analysis was not performed.

### 2.1 Search Strategy and Information Sources

A comprehensive literature search was performed across major scientific databases accessible through Monash University Library: Scopus, Web of Science, PubMed, and ScienceDirect. The search was conducted in April 2025 to capture the most recent publications. No date restrictions were applied to ensure the inclusion of foundational studies. Seminal earlier studies and relevant patents were deliberately included to contextualize the historical evolution of the filed.

The search strategy combined keywords representing three core concepts—SLs, lipase stability, and substrate characteristics, using Boolean operators (AND, OR). Some related terms such as “MLCT”, “Medium‐ and long‐chain triacylglycerol”, “human milk fat substitutes”, and “diacylglycerols” were included. Lipase stability was captured using keywords such as “operational stability”, “half‐life”, “deactivation rate constant”, “reusability”, and “recyclability”. For substrate characteristics, terms such as “oxidation products”, “aldehydes”, “peroxides”, “free fatty acid”, “acyl donor”, “moisture content”, and “water activity” were applied to broaden the search scope.

Subsequently, studies were screened for eligibility based on predefined inclusion and exclusion criteria. Studies were included if they were original research articles or peer‐reviewed review papers, focused on the enzymatic synthesis of SLs, lipase operational stability, deactivation mechanisms, or the influence of substrate characteristics on enzyme performance, and were published in English. In contrast, studies were excluded if they were conference abstracts, editorials, or non–peer‐reviewed sources, lacked relevant quantitative or qualitative data on lipase stability or substrate‐related effects, or were not related to SLs synthesis or lipase‐catalyzed reactions in lipid‐based systems.

**FIGURE 1:**
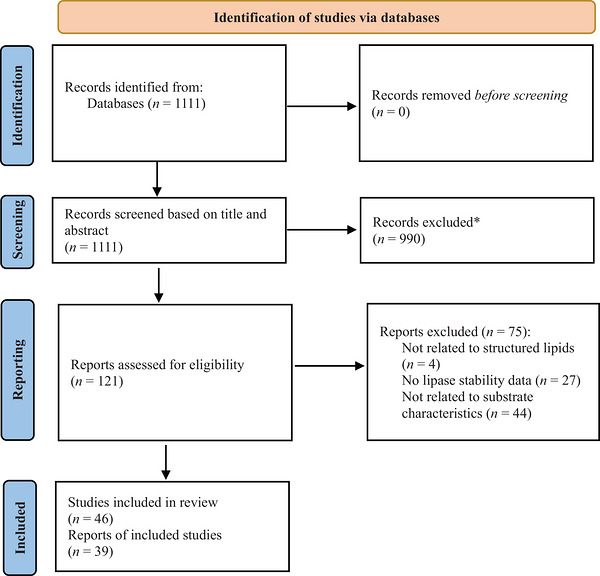
PRISMA 2020 flow diagram illustrating the literature search, screening, and selection process.

The retrieved articles were screened based on titles, abstracts, and full texts to assess eligibility, only studies meeting the inclusion criteria were retained for qualitative synthesis. Reference lists of selected articles were also screened to identify additional relevant studies. The reasons for exclusion at the full‐text stage were recorded and are summarized in the PRISMA flow diagram (Figure 1).

From the included studies, the following data were extracted: author(s), year of publication, lipase type, immobilization strategy, substrate composition and quality attributes, reaction conditions, and reported stability metrics (e.g., half‐life, deactivation rate constant). Given the heterogeneity of study designs, reaction systems, and reported stability metrics, a qualitative synthesis (critical review) was conducted rather than a meta‐analysis. Findings were thematically organized around substrate‐related stressors and corresponding mitigation strategies.

## 3 Lipase Structure and Mechanism

Lipases (EC 3.1.1.3) are serine hydrolases that catalyze the hydrolysis and transesterification of ester bonds in TAGs and other lipid substrates. Lipase's catalytic function and substrate specificity are tightly governed by their three‐dimensional structure, which is stabilized by hydrogen bonds, disulfide bridges, and hydrophobic interactions. It possesses catalytic triad, composed of a nucleophilic residue (usually serine, although cysteine or aspartate may also be present), an acidic residue (aspartate or glutamate), and a histidine residue. The spatial arrangement of these residues facilitates nucleophilic attack on the ester bond of the substrate during catalysis. Crucially, lipase activity is regulated by a flexible lid domain, a surface loop that covers the active site in the inactive conformation. Upon contact with lipid–water interface, the lid undergoes a conformational change, exposing the active site in a process known as interfacial activation. This mechanism enhances substrate recognition, orientation, and catalytic efficiency. Lipases are further adapted to accommodate lipids with varying chain lengths, unsaturation levels, and stereochemistry, providing high substrate specificity. Disruption of these structures, whether due to mutations, or external stressors, can compromise enzyme function (Casas‐Godoy et al. 2012; Jaeger et al. 1994; Peng et al. 2020).

**FIGURE 2:**
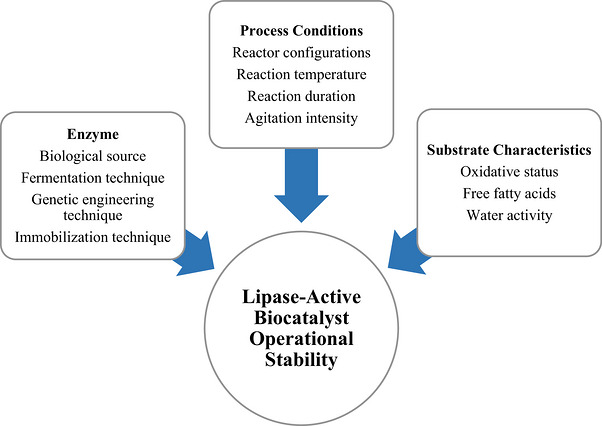
Factors affect operational stability of lipase‐active biocatalyst.

As summarized in Figure 2, operational stability of lipase is influenced by three main factors: (i) enzyme‐related properties, (ii) enzymatic process conditions, and (iii) substrate characteristics. The former two factors have been extensively investigated and reviewed by various research groups (Almeida et al. 2021; Bose et al. 2024; Filho et al. 2019; Liu et al. 2020; Wang et al. 2025). Despite lipases’ sensitivity to substrate characteristics, the latter has not been a primary focus area. Degraded lipid substrates, through oxidation, hydrolysis, or contamination, can alter the lipid–water interface, impede lid opening, and reduce enzyme activity. For instance, lipid oxidation products such as aldehydes and peroxides may introduce polar or bulky groups that interfere with lid displacement and hydrophobic interactions. Moreover, these reactive species can modify key amino acid residues within the enzyme, leading to partial unfolding, active site distortion, or irreversible inactivation (Barbe et al. 2009; Maruyama et al. 2000). These structure–function dependencies highlight the vulnerability of lipases to substrate‐induced stress.

## 4 Lipid Substrates

SLs are typically synthesized via enzymatic modifications such as interesterification, acidolysis, and esterification, which enable the alteration of acylglycerol structures. The use of lipases offers several advantages over chemical methods, including high regioselectivity, mild reaction conditions, lower energy requirements, and greater substrate flexibility. Table 1 summarizes the typical substrates, products and by‐products involved in SL synthesis. A broad range of lipid substrates can be used in SL production, including TAG, DAG, monoacylglycerols (MAGs), glycerol, FFA, fatty acid methyl esters (FAMEs), and fatty acid ethyl esters (FAEEs). The selection of an appropriate substrate depends on multiple factors such as the targeted lipid structure, substrate cost and availability, and the requirements for downstream product purification.

**TABLE 1:** Typical lipid substrates, main products, and by‐products involved in structured lipid biosynthesis.

Reaction	Lipid substrates	Main products	By‐products
Interesterification	TAG + TAG TAG + DAG TAG + MAG TAG + FAME TAG + FAEE	Structured TAGs	Unreacted TAG DAG MAG FFA Residual FAME Residual FAEE
Acidolysis	TAG + FFA	Structured TAG	Unreacted TAG DAG MAG FFA
Esterification	Glycerol + FFA DAG + FFA MAG + FFA	Structured MAG, DAG, or TAG	Water Unreacted FFA DAG MAG

Abbreviations: DAG, diacylglycerol; FAEE, fatty acid ethyl ester; FAME, fatty acid methyl ester; FFA, free fatty acids; MAG, monoacylglycerol; TAG, triacylglycerol.

However, the choice of substrate also has significant implications for lipase performance and stability. Certain substrates and reaction by‐products, such as FFA and water, can accumulate during processing and interact unfavorably with the enzyme. Besides, PUFA substrates such as DHA (C22:6), docosapentaenoic acid (DPA, C22:5), EPA (C20:5), arachidonic acid (ARA, C20:4), and alpha linolenic acid (ALA, C18:3) are especially susceptible to oxidative degradation due to their high degree of unsaturation. Even when refined, bleached, and deodorized oils are used to reduce oxidation, these oils may still undergo gradual degradation over time. The resulting oxidative by‐products, such as hydroperoxides and aldehydes, can compromise lipase stability. It should be noted that the incorporation of PUFAs (in the form of fish oil, seal blubber oil, or single cell oil), into SLs is highly desirable (Table 2); however, as aforementioned, their instability may pose a significant challenge during enzymatic processing (Ibrahim et al. 2008; Osório et al. 2006).

**TABLE 2:** Structured lipids synthesis involving high‐value lipid substrates and nontraditional lipid sources.

	Lipid substrates	Biocatalyst	References
High‐value lipid substrates	**Fish oil** + sunflower oil	*Lipozyme TL IM*	Ibrahim et al. (2008)
	**Menhaden fish oil** + caprylic acid	*Lipozyme IM*	Akoh and Moussata (2001)
	**Menhaden fish oil** + caprylic acid	*Novozyme 435*	Lee et al. (2021)
	**Tuna oil** + caprylic acid	*Lipase Rd (Rhizopus delemar)* *Lipase D (Rhizopus oryzae)* *Lipase AK (Pseudomonas fluorescens)*	Hita et al. (2007)
	**Seal blubber oil** + lauric acid	*Lipozyme IM* *Novozyme 435* *Lipase PS‐30*	Senanayake and Shahidi (2007)
	**EPA‐rich fish oil** + medium‐chain triacylglycerol	*Lipozyme RM IM*	Wang et al. (2022)
	**Algal oil** + lauric acid	*Lipozyme RM IM* *Lipozyme TL IM* *Lipozyme AOAB8*	Li et al. (2021)
	**Arachidonic acid‐rich single‐cell oil (ARASCO)** + tripalmitin	*Lipozyme RM IM*	Liu et al. (2017)
	***Aurantiochytrium limacinum SR21* oil** + caprylic acid	*Lipozyme RM IM* *Lipozyme TL IM*	Delgado Naranjo et al. (2021)
	***Schizochytrium* sp. oil** + medium‐chain triacylglycerol	*NS40086* *Novozyme 435* *Lipozyme RM IM* *Lipozyme TL IM*	Zou et al. (2021)
	***Schizochytrium* sp. *oil*** + oleic acid	*Lipozyme RM IM*	Wang et al. (2015)
	***Isochrysis galbana* oil** + caprylic acid	*Lipozyme TL IM*	He et al. (2018)
	**Algal oil** + medium‐chain triacylglycerol ***Malania oleifera* oil *+*** + medium‐chain triacylglycerol	*Lipozyme RM IM* *Lipozyme TLIM* *Novozyme 435* *Candida* sp. lipase *(CSL)* @ hydrophobic hollow mesoporous silica spheres (HHSS)	Lai et al. (2023)
Nontraditional lipid sources	**Cold‐pressed grapeseed oil** + capric acid/ethyl caprate **Apricot kernel oil** + capric acid/ethyl caprate **Milk thistle oil** + capric acid/ethyl caprate	*Lipozyme TL IM*	Akbaş et al. (2025)
	**Grapeseed oil** + capric acid	*Lipozyme RM IM*	Cozentino et al. (2022)
	**Crude olive pomace oil** + caprylic acid/ethyl caprylate **Crude olive pomace oil** + capric acid/ethyl caprate	*Lipozyme RM IM* *Lipozyme TL IM*	Heinzl et al. (2022)
	**Crude olive pomace oil** + caprylic acid/ethyl caprylate **Crude olive pomace oil** + capric acid/ethyl caprate	*Lipozyme RM IM* *Lipozyme TL IM*	Souza‐Gonçalves et al. (2023)
	**Spent coffee ground oil** + caprylic acid/ethyl caprylate **Spent coffee ground oil** + capric acid/ethyl caprate	*Lipozyme RM IM* *Lipozyme TL IM*	Mota et al. (2020)
	***Cinnamomum camphora* seed oil + camellia oil**	*Lipozyme RM IM*	Zhao et al. (2014)
	**Argan oil** + caprylic acid or capric acid	*Lipozyme RM IM* *Lipozyme TL IM* *Novozyme 435*	Simões et al. (2021)
	**Silkworm (*Bombyx mori* L.) pupae oil** + oleic acid	*Lipozyme RM IM*	Wang et al. (2019)

The product obtained will be structured lipid.

In parallel, growing economic and environmental pressures have driven interest in using nonconventional lipid sources, including crude oils, industrial side‐streams, food processing by‐products, and underexplored materials (i.e., crude olive pomace oil, spent coffee ground oil, milk thistle oil, argan oil; Table 2) for SLs synthesis. While these feedstocks offer advantages in terms of cost reduction and waste valorization, they often contain impurities such as residual FFAs, oxidized lipids, moisture, and other degradation compounds. These impurities may interact adversely with lipases, leading to active site blockage, structural destabilization, or even irreversible enzyme deactivation, ultimately reducing operational lifespan of the biocatalyst and increasing process variability.

Therefore, understanding the composition and quality of lipid substrates is critical for maintaining enzyme performance in SLs synthesis. The following sections will explore how specific substrate characteristics influence lipase operational stability and possible mitigation strategies to enhance lipase operational stability.

## 5 Influence of Lipid Substrate Characteristics on Operational Stability of Lipase

### 5.1 Oxidative Status of Lipids

Lipid oxidation is a free radical‐mediated chain reaction that generates a complex array of degradation products: primary oxidation products such as hydroperoxides and secondary products such as aldehydes, ketones, and polymers, whose diversity increases with the number of double bonds present (Jacobsen et al. 2021). These oxidation products compromise both the sensory attributes and nutritional value of lipids and pose cytotoxic risk (Esterbauer 1993; Moumtaz et al. 2019). In enzymatic SL synthesis, the presence of oxidized lipid substrates introduces additional complexity by impairing lipase operational stability (Table 3). While the implications of lipid oxidation on food quality are well recognized, its influence on lipase biocatalytic processes has received less scrutiny, and the available data reveal substantial methodological heterogeneity that limits cross‐study comparison.

**TABLE 3:** Summary of studies evaluating the effect of lipid oxidative status on lipase operational stability in structured lipid synthesis.

Substrates	Biocatalyst	Reactor	Duration (h)	PV (mEq/kg)	Carbonyl value (mEq/kg)	Abs_232 nm_	Abs_270 nm_	TBARS (mmol TBARS/kg)	*p*‐AnV	Operational stability in half‐life (h)	References
Safflower oil + glycerol	Pseudomonas fluorescens	Continuous membrane bioreactor	180	0 100	2.2–2.5 2.2–2.5					n.d. ∼80	Ohta et al. (1989)
Triolein + lauric acid in petroleum ether	Mucor meihei, support not disclosed (Lipozyme IM20)	Batch	100	0 50						n.d. 80	Wang and Gordon (1991)
Geraniol + ethyl caprate in petroleum ether	*Novozyme 435*	Packed‐bed reactor	80	< 20 < 20				< 20 ∼300		> 72 ∼10	Pirozzi (2003)
High‐oleic sunflower oil + stearic acid in hexane and ethanol	Alcaligenes, support not disclosed (Lipase PL)	Continuous, reactor	Not disclosed	15.5 36.9					1.5 9.1	1053 292	Nezu et al. (1994)
Palm stearin + palm kernel oil + EPAX 4510TG palm stearin + palm kernel oil + sunflower oil	*Lipozyme TL IM*	Packed‐bed reactor	390 580			6.69 5.24	0.74 0.67		0.41 0.44	77 135	Osório et al. (2006)
Cold‐pressed grapeseed oil + ethyl caprate Cold‐pressed grapeseed oil + capric acid	*Lipozyme TL IM*	Packed‐bed reactor	160 150	14.05 14.05						n.d. n.d.	Akbaş et al. (2025)
Sunflower oil + fish oil	*Lipozyme TL IM*	Packed‐bed reactor	200	< 5						187	Ibrahim et al. (2008)
Palm stearin + soybean oil	*Novozyme 435*	Fluidized‐bed reactor	504			< 0.2	< 0.7			408	Osório et al. (2005)
Palm stearin + palm kernel oil + virgin and refined olive oil	*Lipozyme TL IM* *Lipozyme RM IM*	Packed bed reactor	226 188			1.7–2.4 1.7–2.4	0.5–1.1 0.5–1.1			88 60	De Martini Soares et al. (2013)

Half‐life values reported as “n.d.” indicate nondetectable deactivation throughout the experiment timeframe. Lipozyme TL IM is immobilized *Thermomyces lanuginosa* supported on silica granulation; Lipozyme RM IM is immobilized *Rhizomucor meihei* supported on microporous anion exchange resin; *Novozyme 435* is immobilized Lipase B from *Candida antarctica* supported on microporous acrylic resin (LEWATIT P OC 1600).

#### 5.1.1 Primary Oxidation Markers and Their Limitations

The first reported link between lipid oxidation and enzyme deactivation was made by Ohta et al. (1989), who studied the glycerolysis of safflower oil catalyzed by free *Pseudomonas fluorescens* lipase. Substrates with high peroxide value (PV ≈ 100 milliequivalents of active oxygen per kilogram of fat, mEq/kg) resulted in marked reductions in initial catalytic activity and long‐term enzyme stability, with a reported half‐life of 80 h. In contrast, substrates with negligible PV showed no observable deactivation. The authors attributed enzyme instability to hydroperoxide‐induced enzyme modification (Ohta et al. 1989).

In contrary, Wang and Gordan (1991) found that substrates with high PV (∼50 mEq/kg) had little immediate effect on lipase activity, yet repeated batch cycles led to a 50% loss in enzyme stability within 80 h in the acidolysis of triolein with lauric acid using immobilized lipase *Lipozyme IM20*. This suggested that while hydroperoxides initiate the degradation, their decomposition products, likely reactive radicals or secondary carbonyls, play a more critical role in enzyme destabilization (Wang and Gordon 1991).

More recent studies highlight the inadequacy of PV as a sole indicator. Akbaş et al. (2025) studied continuous acidolysis and interesterification of cold‐pressed grapeseed oil with capric acid and ethyl caprate using *Lipozyme TL IM* lipase. Despite a moderate PV of 14.05 mEq/kg, enzyme stability remained stable over 150–160 h (Akbaş et al. 2025). Conversely, Ibrahim et al. (2008) reported 50% enzyme deactivation after 187 h of interesterification using refined sunflower and fish oils in packed‐bed reactor (PBR) with considerably lower PV (< 5 mEq/kg). The introduction of a precolumn with adsorbents, placed between the substrate reservoir and PBR markedly improved lipase stability, implicating secondary oxidation products or other stressors, undetectable by PV alone, as the main contributors to enzyme deactivation (Ibrahim et al. 2008).

These mixed findings indicate that while PV offers a useful indication of early lipid oxidation, it does not fully capture the oxidative burden affecting enzyme performance. A more comprehensive understanding of the lipid oxidative profile is necessary, especially under conditions involving long‐term or repeated lipase use.

#### 5.1.2 Secondary Oxidation Markers and Their Greater Impact

A growing body of research highlights that secondary oxidation products, particularly reactive aldehydes, exert a more severe effect on lipase stability than primary hydroperoxides. Pirozzi (2003) demonstrated that inactivation of *Novozyme 435* was most pronounced in substrates containing high levels of thiobarbituric acid reactive substances (TBARS), a marker of aldehyde accumulation, even when PVs remained low (< 20 mEq/kg). Under high‐TBARS conditions (∼300 mmol TBARS/kg), the enzyme half‐life dropped to only 10 h, whereas under low‐TBARS (< 20 mmol TBARS/kg) and low‐PV (< 20 mEq/kg) conditions, half‐life exceeded 72 h. Specific aldehydes derived from ω‐6 and ω‐3 fatty acids, such as hexanal, 4‐hydroxy‐2‐nonenal (4‐HNE), malondialdehyde (MDA), and 4‐hydroxy‐2‐hexenal (4‐HHE), were identified as potent inhibitors, underscoring the direct role of secondary oxidation in lipase deactivation (Pirozzi 2003).

Nezu et al. (1994) further substantiated these findings by correlating enzyme deactivation rate (expressed as the deactivation constant *k*_d_) with both primary and secondary oxidation markers. Oils exhibiting higher values (PV = 36.9 mEq/kg; *p*‐AnV = 9.1) showed faster enzyme degradation compared to oils with lower values (PV = 15.5; *p*‐AnV = 1.5). This emphasizes that primary and secondary oxidation products act cumulatively to accelerate lipase inactivation, and relying solely on PV is insufficient to predict operational stability (Nezu et al. 1994).

Spectrophotometric UV absorbance at 232 nm and 270 nm, indicating conjugated dienes (primary oxidation) and carbonyl compounds (secondary oxidation), respectively, provides a complementary approach to assess oxidative status. Studies employing *Lipozyme TL IM* in PBR reveal a clear inverse relationship between UV absorbance values and lipase half‐life. De Martini Soares et al. (2013) reported moderate oxidation levels (Abs_232_ 1.7–2.4; Abs_270_ 0.5–1.1) and a corresponding half‐life of 88 h. In contrast, Osório et al. (2006) observed substantially higher primary oxidation (Abs_232_ 5.24–6.69) when processing substrates containing PUFAs, with half‐lives of 135 h (sunflower oil) and 77 h (EPAX 4510TG, rich in EPA/DHA; Osório et al. 2006; de Martini Soares et al. 2013). Notably, under identical enzyme and reactor conditions, the more unsaturated substrate yielded the highest Abs_232_ (6.69) and the shortest half‐life (77 h), illustrating that substrate unsaturation directly amplifies oxidative stress and accelerates enzyme deactivation.

Reactor configuration also affected the oxidation‐related stability. Osório et al. (2005) reported an outstanding half‐life of 408 h for *Novozyme 435* in a fluidized‐bed reactor (FBR) using palm stearin and soybean oil, with Abs_232_ maintained below 0.2 and Abs_270_ below 0.7. This combination of low‐oxidation substrate, reduced mechanical stress, and favorable enzyme–substrate compatibility collectively contributes to extended operational stability (Osório et al. 2005).

**FIGURE 3:**
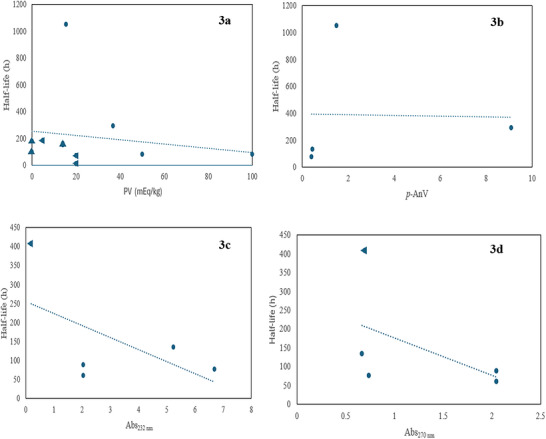
Relationship between half‐life (h) and oxidation‐related parameters during structured lipid synthesis: (a) peroxide value (PV, mEq/kg), (b) *p*‐anisidine value (*p*‐AnV), (c) absorbance at 232 nm (Abs_232 nm_), and (d) absorbance at 270 nm (Abs_270 nm_). Circles represent experimentally determined half‐life values. Upward‐pointing triangles indicate cases where no deactivation was observed within the experimental timeframe (i.e., half‐life exceeds the experiment duration). Sideward‐pointing triangles denote data points obtained at the upper limit of the parameter range investigated. Dashed lines represent linear regressions. Data points are compiled from Ohta et al. (1989), Wang and Gordon (1991), Pirozzi (2003), Nezu et al. (1994), Akbaş et al. (2025), Ibrahim et al. (2008), Osório et al. (2006), Osório et al. (2005), and De Martini Soares et al. (2013).

Collectively, these studies reinforce that secondary oxidation products, particularly reactive aldehydes, are critical drivers of lipase deactivation. However, cross‐study comparisons remain severely limited by methodological heterogeneity, as illustrated in Figure 3. Studies employ these markers interchangeably despite their measurement of different oxidation stages. Additionally, the inconsistencies in reactor configurations, enzyme types and reporting practices further complicated the difficulty of drawing quantitative comparisons. A multiparameter assessment, alongside consideration of substrate composition, lipase characteristics, and reactor design, are essential for accurately evaluating substrate suitability and predicting biocatalyst operational stability.

### 5.2 Mechanism of Lipase Deactivation by Oxidized Lipid Substrates

The deactivation of lipases by oxidized lipid substrates is driven by a combination of chemical modification and physical fouling, both of which contribute to the decline in lipase catalytic performance over time.

#### 5.2.1 Chemical Modification and Structural Damage

**FIGURE 4:**
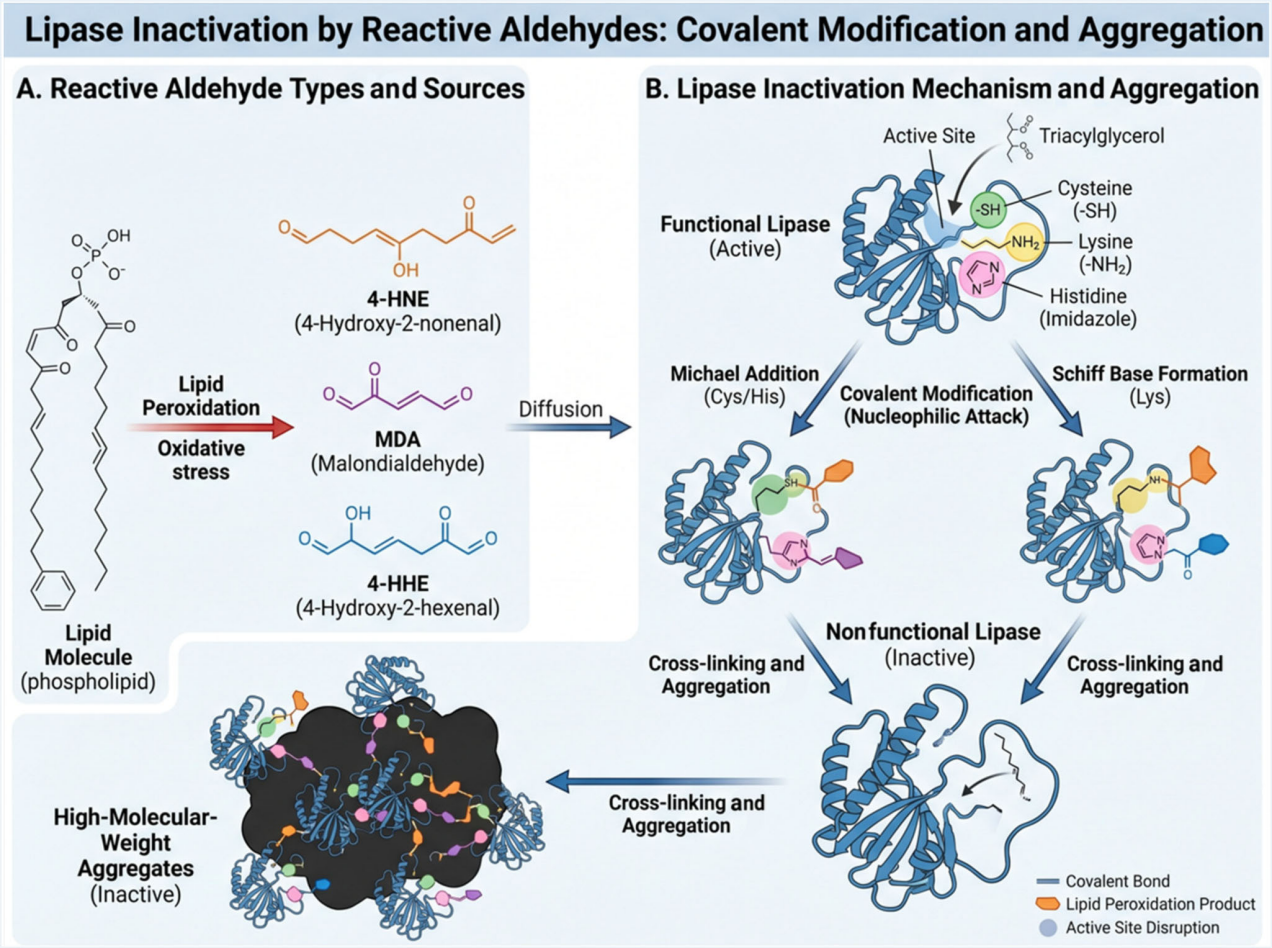
Mechanism of lipase deactivation by oxidized lipid substrates through chemical modification and structural damage. Schematic elements in this figure were created using AI‐assisted tools and subsequently reviewed and edited by the authors for accuracy.

Reactive aldehydes, particularly 4‐HNE, MDA, and 4‐HHE, irreversibly inactivate lipases by covalently binding to nucleophilic amino acid residues, especially cysteine (–SH), lysine (–NH_2_), and histidine (imidazole) side chains, through mechanisms such as Michael addition or Schiff base formation. These residues are often located near or within the enzyme's active site, making them especially vulnerable. Covalent modifications disrupt catalytic triad geometry, alter enzyme conformation, induce enzyme aggregation and cross‐linking, and promote formation of inactive high‐molecular‐weight aggregates (di Lorenzo et al. 2007; Ohta et al. 1989; Pirozzi et al. 2023; Xu et al. 1998; Figure 4).

Supporting this, Pirozzi (2003) pretreated *Novozyme 435* with p‐hydroxymercuribenzoate (pHMB), a thiol‐blocking reagent that protects cysteine residues. When subsequently exposed to 4‐HNE and MDA, the pretreated lipase retained significantly higher activity compared to untreated controls, directly implicating cysteine modification resulting in aldehyde‐induced inactivation (Pirozzi 2003). Additionally, early work by Ohta et al. (1989) revealed that exposure to lipid hydroperoxides induced lipase polymerization in *P. fluorescens*, as observed via SDS‐PAGE. The resulting high‐molecular‐weight aggregates were enzymatically inactive, revealing that hydroperoxide‐driven polymerization is an irreversible pathway for enzyme deactivation (Ohta et al. 1989).

#### 5.2.2 Physical Fouling and Diffusion Limitations

Beyond chemical modification, oxidized lipid substrates can cause noncovalent physical fouling of the enzyme, particularly in immobilized systems. Oxidized products, such as polymerized TAGs, polar aldehydes, and lipid‐derived pigments, can adsorb to the surface of immobilization matrix. This adsorption may block substrate access to the active site, impede mass transfer, or alter the microenvironment surrounding the enzyme, leading to reduced catalytic efficiency. This issue is especially pronounced in long‐term processing operations or when using minimally processed or unrefined oils are used.

Xu et al. (1998) evaluated the reversibility of lipase inhibition by various oxidized lipid components. Partial recovery of enzyme activity (20%–40%) was achieved after solvent washing, especially in cases involving hydroperoxides or polymerized lipids. In contrast, inhibition caused by carotenoids, believed to adsorb physically without covalent modification, was largely reversible (Xu et al. 1998). This distinction underscores the importance of differentiating between reversible fouling and irreversible chemical damage. Similarly, Osório et al. (2005) demonstrated that lipase stability lost during continuous interesterification could be partially restored with a triple hexane wash, allowing enzyme reuse for an additional 13 days (Osório et al. 2005). These findings suggest that fouling‐induced activity loss, while detrimental, can be reversed through appropriate cleaning protocols.

### 5.3 Free Fatty Acid Composition

In the enzymatic synthesis of SLs, FFAs serve either as intentional acyl donors in acidolysis and esterification reactions or as transient intermediates during interesterification. The molecular characteristics (e.g., chain length, polarity) and their role within the reaction system impact lipase operational stability.

#### 5.3.1 Influence of Acyl Donors Form and Chain Length on Lipase Stability

**FIGURE 5:**
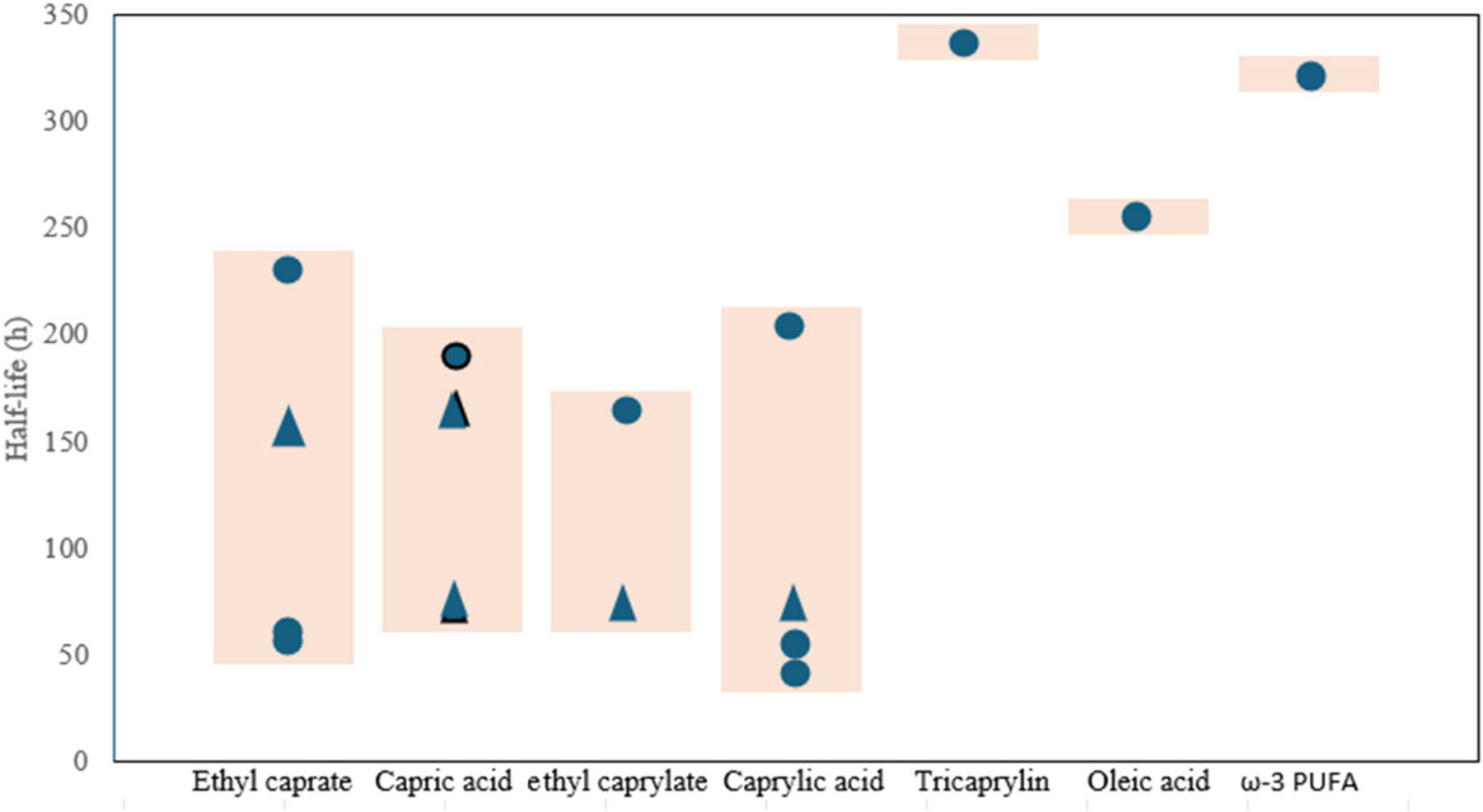
Half‐life (*h*) of lipase under structured lipid synthesis when different fatty acid donors were used. Circles correspond to experimentally determined half‐life values. Triangles indicate cases where no deactivation was observed over the course of the experiment, and thus the half‐life exceeds the experimental duration. Shaded regions represent the range of observed values. Data points are compiled from Heinzl et al. (2022), Souza‐Gonçalves et al. (2023), Mota et al. (2020), Akbaş et al. (2025), Utama et al. (2020), Nunes et al. (2012), and Tecelão et al. (2010).

The studies summarized in Table 4 demonstrate that lipase operational stability is governed by a complex interplay between acyl donor characteristics, reaction system, and biocatalyst. Among these factors, the chain length of the acyl donor and structural configuration molecular emerge as primary determinants of enzyme deactivation (Figure 5).

**TABLE 4:** Summary of studies evaluating the effect of fatty acid donor form and chain length on lipase operational stability in structured lipid synthesis.

Long‐chain fatty acid donor	Medium‐chain fatty acid donor	Biocatalyst	Reactor	Synthesis duration (h)	FFA (%)	Operational stability in half‐life (h)	References
Crude olive pomace oil	Ethyl caprylate Ethyl caprate Caprylic acid Capric acid	*Lipozyme RM IM*	Batch	91	3.4 20 3.4 20 3.4 20 3.4 20	n.d. 163 n.d. 189 108 51 n.d. 57	Heinzl et al. (2022)
Crude olive pomace oil	Ethyl caprylate Ethyl caprate Caprylic acid Capric acid Ethyl caprylate Ethyl caprate Caprylic acid Capric acid	*Lipozyme RM IM* *Lipozyme TL IM*	Packed‐bed reactor	70	28.75 12.05 28.75 12.05 28.75 12.05 28.75 12.05	74 n.d. n.d. n.d. n.d. n.d. n.d. 228	Souza‐Gonçalves et al. (2023)
Spent coffee ground oil	Ethyl caprylate Ethyl caprate Caprylic acid Capric acid	*Lipozyme RM IM*	Batch	70	4.6 4.6 4.6 4.6	n.d. n.d. 47 54	Mota et al. (2020)
Cold‐pressed grapeseed oil	Ethyl caprate Capric acid	*Lipozyme TL IM*	Packed‐bed reactor	160 150	Not indicated Not indicated	n.d. n.d.	Akbaş et al. (2025)
Palm olein	Tricaprylin Caprylic acid	*Lipozyme TL IM*	Batch	120	Not indicated Not indicated	336 204	Utama et al. (2020)
Virgin olive oil	Caprylic acid Capric acid	Heterologous *R. oryzae* immobilized on Eupergit C	Batch	299	Not indicated	39.0 54.3	Nunes et al. (2012)
Tripalmitin	Oleic acid ω‐3 PUFA	*Lipozyme RM IM* *Lipozyme TL IM* *Novozyme 435* *C. parapsilosis* immobilized on AccurelMP1000 *Lipozyme RM IM* *Novozyme 435* *C. parapsilosis* immobilized on AccurelMP1000	Batch	230	Not indicated	n.d. 154 253 276 34.5 322 127	Tecelão et al. (2010)

Half‐life values reported as “n.d.” indicate nondetectable deactivation throughout the experiment timeframe. Lipozyme TL IM is immobilized *Thermomyces lanuginosa* supported on silica granulation; Lipozyme RM IM is immobilized *Rhizomucor meihei* supported on microporous anion exchange resin; *Novozyme 435* is immobilized Lipase B from *Candida antarctica* supported on microporous acrylic resin (LEWATIT P OC 1600).

A consistent trend across multiple system is the pronounce destabilizing effect of medium‐chain FFAs, particularly, caprylic acid (C8:0), compared to longer chain FFAs such as capric acid (C10:0). In batch systems using *Lipozyme RM IM*, shorter half‐lives were reported in the presence of C8:0 relative to C10:0 in both crude olive pomace oil and spent coffee ground oil systems (Heinzl et al. 2022; Mota et al. 2020). Similar observations were made with heterologous *R. oryzae* lipase, where half‐life during acidolysis was lower with C8:0 (39 h) than with C10:0 (54 h; Nunes et al. 2012).

In contrast, esterified acyl donors such as ethyl caprylate, ethyl caprate, and tricaprylin generally result in improved enzyme stability compared to their FFA counterparts. Mota et al. (2020) reported no significant deactivation in interesterification systems using ethyl esters, whereas acidolysis systems using FFAs under similar conditions exhibited half‐lives of only 47–54 h (Mota et al. 2020). Interesterification involving TAG substrates also provided satisfactory protection to the lipase. Utama et al. (2020) found that *Lipozyme TL IM* achieved a half‐life of 336 h with tricaprylin compared to 204 h with caprylic acid for the synthesis of SL with refined palm olein (Utama et al. 2020). Likewise, sustained operational stability (half‐lives of 163 and 189 h) has been reported in interesterification systems when ethyl caprylate and ethyl caprate were used respectively, despite the presence of up to 20% FFAs in the reaction mixture (Heinzl et al. 2022). Conversely, Souza‐Gonçalves et al. (2023) reported no noticeable lipase deactivation in either interesterification or acidolysis over a 70‐h period in PBR, though this shorter timeframe may not have been sufficient to capture the full extent of enzyme degradation (Souza‐Gonçalves et al. 2023).

Process configuration further modulates these effects. PBRs generally exhibit enhanced operational stability compared to batch systems, as demonstrated by prolonged enzyme lifetimes in continuous systems (Akbaş et al. 2025; Souza‐Gonçalves et al. 2023). However, direct comparisons remain challenging due to differences in residence time, substrate composition, and FFA concentration.

Lipase species and immobilization matrix also critically influence enzyme stability. Differences observed between *Lipozyme RM IM* (*R. meihei* on microporous anion exchange resin), *Lipozyme TL IM* (*Thermomyces lanuginosus* on silica granules), and *R. oryzae* on Eupergit C to varying acyl donors (Heinzl et al. 2022; Souza‐Gonçalves et al. 2023; Nunes et al. 2012). Moreover, the fatty acid composition of the substrate introduces additional complexity. Tecelão et al. (2010) examined the stability of three immobilized lipases in ten consecutive batch reactions with different acyl donors. *Lipozyme RM IM* exhibited no detectable deactivation with oleic acid (C18:1), but its half‐life decreased to 276 h when exposed to ω‐3 PUFAs. In contrast, *Novozyme 435* and *Lipozyme TL IM* showed the opposite trend: both were more stable with ω‐3 PUFAs (half‐life of 322 h for *Novozyme 435* and no detectable deactivation over 230 h for *Lipozyme TL IM*) than with C18:1 (half‐lives of 253 h and 154 h, respectively; Tecelão et al. 2010). These contrasting behaviors suggest that lipase stability depends not only on fatty acid chain length but also on the degree of unsaturation, and that the effect is enzyme‐specific, likely mediated by differences in the active site architecture and the immobilization support's interaction with polar or oxidized species. Despite these observations, systematic studies isolating the effect of lipase species and immobilization characteristics remain limited.

#### 5.3.2 FFA as Transient Intermediates in Interesterification

FFAs may also form transiently as hydrolytic intermediates in enzymatic interesterification. The mechanism involves sequential hydrolysis and re‐esterification steps, during which FFAs briefly accumulate before being consumed. This is particularly common with immobilized lipases, where residual moisture in the support matrix promotes initial hydrolysis (Osório et al. 2009; Paula et al. 2015; de Martini Soares et al. 2013).

For example, de Martini Soares et al. (2013) reported that in a PBR using palm stearin, palm kernel oil, and olive oil, FFA levels ranged from 3.8% to 4.9% within the first hour. These values dropped to ∼1% within 24 h and remained stable thereafter (de Martini Soares et al. 2013). Importantly, this temporary FFA surge did not impair enzyme activity, likely because the FFAs formed were long‐chain, less polar species that do not destabilize lipases to the same extent as medium‐chain FFAs. Hence, transient FFA accumulation in interesterification systems does not typically compromise enzyme stability, especially when long‐chain FFAs dominate.

#### 5.3.3 Residual Acidity

Beyond the characteristics of FFAs themselves, residual acidity in the lipid substrate is a closely related factor influencing enzyme stability. During oil refining, residuals inorganic acids from degumming or deodorization, or acidic residues from bleaching earth, can remain in the final feedstock. These acidic residues can dissolve in trace amounts of water (< 0.2%), leading to a localized pH drop in the enzyme microenvironment. Such shifts in internal pH, especially in immobilized systems with hydrophilic supports, may promote enzyme deactivation by disrupting hydrogen bonding or electrostatic interactions critical for maintaining enzyme conformation (Ferreira and Tonetto 2017).

Interesterification systems consistently yield longer lipase half‐lives than acidolysis when medium‐chain FFA (e.g., caprylic acid) are used as acyl donor, a trend confirmed across multiple enzymes and reactor configurations. However, the quantitative impact of FFAs on lipase stability varies with lipase type, immobilization matrix properties, acyl donor form, and reaction conditions.

To date, most evidence is derived from a limited set of commercial immobilized lipases (e.g., *Lipozyme RM IM*, *TL IM*), and systematic studies integrating substrate characteristics with immobilization matrix design are lacking, leaving a critical need for predictive models to optimize lipase stability during SLs synthesis within these systems. Future immobilization support designs could be tailored to the expected FFA profile: short‐chain FFAs demand designs that minimize FFA‐enzyme interactions, whereas long‐chain FFAs are less demanding. Predictive models to optimize lipase stability in SL synthesis remain a critical research gap.

### 5.4 Mechanism of Lipase Deactivation by Free Fatty Acids

The inactivation of lipases during acidolysis and esterification reactions has been primarily influenced by the polarity of the FFAs used as acyl donors. A key parameter in this context is the Log *P* value, a partition coefficient that reflects a compound's hydrophobicity. Compounds with lower Log *P* values are more hydrophilic, while those with higher Log *P* values correlate with greater hydrophobicity.

Souza‐Gonçalves et al. (2023) reported that lipase deactivation was inversely related to the Log *P* value of the acyl donors. For example, caprylic acid (C8:0), with a Log *P* of 3.05, exhibited the most significant destabilizing effect on the enzyme, compared to capric acid (Log *P* = 4.09), ethyl caprylate (Log *P* = 3.842), and ethyl caprate (Log *P* = 4.861; Souza‐Gonçalves et al. 2023).

Laane et al. (1987) proposed that compounds with Log *P* values above 4 do not disrupt the hydration shell required for enzyme stability, whereas more polar compounds with Log *P* values below 2 can strip this essential water layer, leading to enzyme deactivation (Laane et al. 1987). Lipases rely on a thin hydration layer of tightly bound water molecules to maintain their active conformation and catalytic functionality. In solvent‐free or low‐water systems, common in enzymatic SLs synthesis, the enzyme's microenvironment is already water‐limited. Under these conditions, polar medium‐chain FFAs like caprylic acid (C8:0) can exacerbate dehydration by interacting with and removing structurally bound water molecules from the enzyme surface. This leads to loss of structural flexibility, distortion of the active site, and eventual decline in catalytic efficiency.

### 5.5 Water Activity

Water is a critical parameter influencing lipase activity, stability, and conformation, and substrate partitioning, as well as the thermodynamic equilibrium of the biocatalysis process. However, its role in long‐term operational stability remains complex and highly system‐dependent. The compiled studies (Table 5) reveal that while controlled water addition often enhances lipase half‐life, the magnitude and even the direction, of the effect is governed by the specific biocatalyst, immobilization support, and the method and amount of water incorporated.

**TABLE 5:** Summary of studies evaluating the effect of water activity on lipase operational stability in structured lipid synthesis.

Lipid substrates	Biocatalyst	Reactor	Synthesis duration (h)	Water (w/w%)	Method of water addition	Operational stability in half‐life (h)	References
Echium oil + butanol	Immobilized *R. oryzae*, support not disclosed	Batch	48	Not added 2.5	Added into substrate that is butanol	24.3 82.5	Corzo‐Martínez et al. (2018)
Virgin olive oil + caprylic acid Virgin olive oil + capric acid Virgin olive oil + capric acid	Heterologous *R. oryzae* immobilized on Eupergit C Heterologous *R. oryzae* immobilized on Lewatit VP OC 1600	Batch	299	Added Added Added Not added	Added by washing recovered lipase with 0.1 M sodium phosphate buffer solution	39.0 54.3 234 49.1	Nunes et al. (2012)
Lard + FFA from EPAX 1050TG	Heterologous *R. oryzae* immobilized on Accurel MP 1000	Batch	168	Water added	Added by washing recovered lipase with 0.1 M sodium phosphate buffer solution	112	Simões et al. (2014)
Palm stearin + palm kernel oil + EPAX 4510TG	*C. parapsilosis* immobilized on Accurel MP 1000	Batch	92	Not added Added to maintain 0.2 equilibrium	Added into substrate	10 18	Osório et al. (2009)
Fish oil + MCT	*Lipozyme TL IM*	Packed‐bed reactor	336	Not added		n.d.	Xu et al. (2002)

Half‐life values reported as “n.d.” indicate nondetectable deactivation throughout the experiment timeframe. Lipozyme TL IM is immobilized *Thermomyces lanuginosa* supported on silica granulation; Lipozyme RM IM is immobilized *Rhizomucor meihei* supported on microporous anion exchange resin; *Novozyme 435* is immobilized Lipase B from *Candida antarctica* supported on microporous acrylic resin (LEWATIT P OC 1600).

#### 5.5.1 Influence of Lipase Source and Immobilization Support on Water Activity

Lipase exhibit different water activity depending on their origin and immobilization status (Xia et al. 2009; Yu et al. 2007). In a systematic evaluation of nonimmobilized lipases in transesterification, Xia et al. (2009) classified enzymes into three categories:

– Type I (low dependence): Activity remains relatively constant across varying *a*_w_ (e.g., *R. niveus*, *R. oryzae*).
– Type II (moderate dependence): Activity fluctuates with *a*_w_; this is the most common group (e.g., *P. fluorescence*, *C. rugosa*, *P. roqueforti*, *P. camemberti*, *A. niger*, porcine pancreases).
– Type III (high dependence): Active only at high *a*_w_ > 0.75 (e.g., wheat germ lipase) (Xia et al. 2009).

Nonimmobilized lipases are generally more sensitive to water content than immobilized lipases, as immobilization modifies the local microenvironment and buffers the enzyme against rapid hydration change (Edlund et al. 1996). For immobilized lipases, water activity dependence is strongly influenced by both the physicochemical properties of the support and the immobilization method (Causevic et al. 2022; Nunes et al. 2012; Páez et al. 2003; Senanayake and Shahidi 2007; Xu et al. 2002; Zhang et al. 2001, 2022).

Hydrophilic supports (e.g., silica gels, Eupergit C) tend to retain water, preserving enzyme flexibility but also increasing the risk of uncontrolled hydrolysis and enzyme leaching, which leads to eventual deactivation (Zhao et al. 2007). Conversely, hydrophobic supports (e.g., Lewatit resins) minimize water accumulation and adsorption of polar by‐products such as glycerol, offering better hydration control under low‐water conditions (Martins et al. 2013; Marty et al. 1997). The immobilization method further modulates hydration tolerance. Covalent binding provides strong attachment but may restrict conformational flexibility and limit water access to the active site. Physical adsorption preserves the native hydration shell more effectively but carries a higher risk of desorption (Nunes et al. 2012). As discussed below, the interplay between support hydrophobicity, immobilization chemistry, and water addition protocol can produce markedly different stability outcomes.

#### 5.5.2 Empirical Evidence: Beneficial Effect and Counter Examples

In several cases, minimal water incorporation substantially improved lipase operational stability. Corzo‐Martínez et al. (2018) reported that supplementing the butanol substrate with 2.5% water extended the half‐life of immobilized *R. oryzae* lipase from 24.3 h to 82.5 h, suggesting that the added water preserved the enzyme's hydration shell and prevented conformational distortion (Corzo‐Martínez et al. 2018). Similarly, Osório et al. (2009) observed a near doubling of half‐life (from 10 h to 18 h) for *C. parapsilosis* lipase immobilized on Accurel MP 1000 when water was introduced to maintain an equilibrium *a*_w_ of 0.2 (Osório et al. 2009).

However, the data also reveal marked inconsistencies that challenge any simple “water‐is‐beneficial” narrative. A particularly instructive case is provided by Nunes et al. (2012), who systematically examined the effects of immobilization support, fatty acid chain length, and water addition protocol on the stability of heterologous *R. oryzae* lipase. When the enzyme was covalently immobilized on the hydrophilic, epoxy‐activated support Eupergit C, rehydration of the biocatalyst between batches with buffer resulted in half‐lives of 39.0 h with caprylic acid (C8:0) and 54.3 h with capric acid (C10:0). In contrast, the same lipase physically adsorbed onto the hydrophobic resin Lewatit VP OC 1600 exhibited a half‐life of 49 h with capric acid when no water was added. Remarkably, when the adsorbed lipase on Lewatit VP OC 1600 was rehydrated by buffer washing between batches, again using capric acid, the half‐life soared to 234 h, more than four times longer than the nonrehydrated control. These contrasting outcomes underscore that the effect of water is not monotonic. Furthermore, the shorter half‐lives observed with the covalently immobilized enzyme (Eupergit C) suggest that the strong attachment may restrict water accessibility to the active site (Nunes et al. 2012).

Further illustrating this complexity, Osório et al. (2009) achieved only modest stability (18 h) with *C. parapsilosis* supported on Accurel MP 1000 even with controlled water addition, whereas Simões et al. (2014) obtained a half‐life of 112 h using the same support but a different lipase (*R. oryzae*) and a comparable buffer‐washing hydration method. This discrepancy highlights that enzyme source and immobilization procedure collectively dictate the final stability outcome (Osório et al. 2009; Simões et al. 2014).

Across the studies, a critical limitation is the absence of measured water activity (*a*_w_). *a*_w_ is the thermodynamically relevant parameter governing enzyme hydration, mass transfer, and reaction equilibrium (Xia et al. 2009). For example, the same nominal 2.5% water added to a nonpolar oil yields a much higher *a*_w_ than when added to a polar alcohol, yet such differences are masked in current reporting. Moreover, water is consumed or produced during esterification, meaning that initial water addition does not guarantee constant hydration throughout the reaction. Real‐time monitoring of *a*_w_ or the use of controlled‐*a*_w_ system (e.g., via saturated salt solution) would enable more reproducible and mechanistically insightful studies. The lack of standardized *a*_w_ measurement severely compromises direct comparison across studies and impedes the development of predictive models for lipase stability under varying moisture conditions.

### 5.6 Mechanistic Insights: Stabilizing and Destabilizing Roles of Water

Collectively, the evidence confirms that water is a double‐edged sword for lipase operational stability. An optimal hydration level is essential to preserve the enzyme structure and activity, but deviations, whether too low or too high, can accelerate deactivation. The following mechanistic studies elucidate both the molecular and physical bases off its effect.

#### 5.6.1 Stabilizing Role

Water contributes to lipase stability primarily through the preservation of the enzyme's essential hydration shell. A minimum layer of bound water is required to maintain the native conformation, ensuring that the catalytic triad remains correctly oriented and that the “lid” structure retains the flexibility necessary for the interfacial activation. Zhang et al. (2022) observed that in a PBR, the moisture content of *Lipozyme TL IM* declined modestly from 6.2% to 5.3% over 100 h of continuous operation, with only 0.5% of free water consumed; the remainder was hypothesized to be tightly bound water essential for catalytic activity (Zhang et al. 2022). Osório et al. (2009) provided direct evidence that added water primarily stabilizes the enzyme active conformation rather than being consumed as a reagent. In their interesterification system, water concentration in the outlet stream consistently exceeded that in the inlet, indicating that the introduced water functioned mainly to maintain hydration of the biocatalyst rather than to participate in hydrolysis (Osório et al. 2009). Interestingly, the amount of bound water required for stability may be lower than typically assumed. Zhang et al. (2001) found that deliberately drying *Lipozyme TL IM* from 6% to 3% moisture had little effect on lipase activity, while Xu et al. (2002) reported sustained *Lipozyme TL IM* stability over 2 weeks without water supplementation (Xu et al. 2002; Zhang et al. 2001). Collectively, these findings suggest that only a tightly bound water fraction, constituting the enzyme's indispensable hydration shell, is critical for maintaining catalytic conformation; excess free water is neither required nor necessarily beneficial.

#### 5.6.2 Destabilizing Role

At elevated concentrations, water induces deactivation through distinct physical and molecular mechanisms (Figure 3). At the physical level, Páez et al. (2003) used mercury porosimetry to demonstrate that increasing water content leads to progressive flooding of carrier pores via capillary adsorption. This process begins with the narrowest pores, which become saturated first, effectively isolating enzyme molecules from the bulk substrates and reducing catalytic efficiency (Páez et al. 2003). Such pore flooding is particularly pronounced in hydrophilic supports, where water is preferentially retained. At the molecular level, Peng et al. (2020) employed molecular dynamics simulations to reveal how excess water disrupts lipase function. While a low‐water content (5% w/w) facilitates stabilizing hydrogen bonds and maintains the hydrophobic environment around the active site, higher water levels introduce excessive water molecules into the catalytic pocket. These water molecules compete with substrate for binding, increase hydrogen bonding with polar residues of the lid and the active site, thereby restricting the conformational flexibility required for interfacial activation and lid opening. Additionally, elevated water content increases the polarity of the reaction medium, raising the activation energy of the biocatalysis and shifting the thermodynamic equilibrium toward hydrolysis. The net effect is a reduction in catalytic turnover and accelerated deactivation (Peng et al. 2020).

In summary, the mechanistic evidence supports a model in which water exerts a narrow optimal window: sufficient bound water is required to preserve the active conformation and enable lid flexibility, but excess water, whether by flooding support pores or by overhydrating the enzyme's active site, leads to physical inaccessibility and molecular rigidity, ultimately compromising lipase stability and activity.

## 6 Mitigation Strategies to Enhance Lipase Operational Stability in Relation to Substrate Characteristics

In industrial biocatalysis, the catalytic efficiency and operational stability of lipases are highly dependent on the physicochemical characteristics of the substrate, as discussed. Although commercially available lipid substrates are typically processed to meet stringent quality standards under good manufacturing practices (GMPs), deviations from these standards may occur due to various practical constraints. These include the use of aged or oxidized lipids, alternative feedstocks driven by supply chain disruptions, or cost‐reduction measures. Under such suboptimal conditions, lipase performance can be significantly impaired, necessitating a focused approach to substrate management as part of the process optimization. Advances in pretreatment techniques, antioxidant supplementation, process design including reactor configuration, reactor types, and in situ removal of inhibitory compounds are among the approaches being explored to enhance lipase robustness under challenging substrate conditions (Table 6).

**TABLE 6:** Mitigation strategies to enhance lipase operational stability in relation to substrate characteristics.

Mitigation strategies	Substrates	Lipase	Stability indicator	Stability without treatment	Stability with treatment	References
Incubation of substrates with protein constituents – 0.2 mM albumin	Soybean oil with 0.1 mM 4‐HNE Soybean oil with 0.1 mM MDA	*Novozyme 435*	Residual lipase activity after 24 h	78.6% 73.5%	83.1% 80.9%	Pirozzi (2003)
Precolumn packed with protein constituents—texturized soy protein Precolumn packed with protein‐constituents—texturized soy protein, Pretreatment of substrates with 0.02% antioxidant tBHQ, and nitrogen sparge	Refined, bleached soybean oil (20% fully hydrogenated soybean oil + 80% soybean oil)	*Lipozyme TL IM*	Half‐life	6 days	17 days 42 days	Binder et al. (2006)
Pretreatment of substrates with silica‐gel chromatography	High‐oleic sunflower oil + stearic acid	*Alcarigenes*, support not disclosed (*Lipase PL*)	*k*_d_	0.0569 day^−1^	0.0059 day^−1^	Nezu et al. (1994)
Precolumn packed with molecular sieves Precolumn packed with activated carbon Precolumn packed with spent *Lipozyme TL IM*	Sunflower oil + fish oil	*Lipozyme TL IM*	Half‐life	187 h	578 h 1386 h 770 h	Ibrahim et al. (2008)
Bleaching with adsorbents: 5 wt% bleaching clay Tonsil 210 FF and 2.5 wt% activated carbon based on the weight of the substrate	*Echium* oil + butanol	Immobilized *R. oryzae*, support not disclosed	Activity slope *k*_d_ *Half‐life*	0.4260 ‐ ‐	0.5102 0.0008 h^−1^ 905 h	Corzo‐Martínez et al. (2018)
Pretreatment of substrates with 5 wt% of cocoa butter (as source of natural antioxidants) to substrates Synthesis performed under nitrogen atmosphere in feeding reservoir	Babassu oil + glycerol	*Burkholderia cepacia* lipase supported on SiO_2_–PVA	Product (monoacylglycerol) yield after 192 h	18.2%	25.2% 24.1%	Teixeira et al. (2014)
Pretreatment of substrates with 5 wt% of cocoa butter (as source of natural antioxidants) to substrates Synthesis performed under nitrogen atmosphere in feeding reservoir	Babassu oil + glycerol	*Burkholderia cepacia* lipase supported on SiO_2_–PVA	Product (monoacylglycerol) yield after 192 h	‐ ‐	31.4% 31.5%	Vilas Bôas et al. (2021)
Retreatment of substrate with 0.02 wt% antioxidant tocopherol to substrates Pretreatment of substrate with 0.02 wt% antioxidant EDTA to substrates	Rapeseed oil + caprylic acid Safflower oil + caprylic acid Fish oil + caprylic acid Fish oil + capric acid Rapeseed oil + caprylic acid Safflower oil + caprylic acid Fish oil + caprylic acid Fish oil + capric acid	*Lipozyme RM IM*	Fatty acid incorporation after 24 h	∼34% ∼34% ∼34.5% ∼35.5%	∼40% ∼36% ∼36% ∼39% ∼36% ∼36% ∼36% ∼38%	Xu et al. (2005)
Reactor configuration: multistage packed‐bed reactors	Fully hydrogenated canola oil + soybean oil	*Lipozyme TL IM*	Half‐life	121.6 h (single‐stage packed‐bed reactor)	165 h (double‐packed‐bed reactor) 198 h (triple‐stage packed‐bed reactor)	Won et al. (2012)
Reactor configuration: Multistage packed‐bed reactors	Grape seed oil + capric acid	*Lipozyme RM IM*	Half‐life	209 h (single‐stage packed‐bed reactor)	235 h (double‐stage packed‐bed reactor)	Cozentino et al. (2022)
Adoption of continuous packed‐bed reactors	Palm stearin + oleic acid	*Aspergillus oryzae* supported on macroporous adsorption resin D3520	Half‐life	24 h (batch stirred‐tank reactor)	140 h (continuous packed‐bed reactor)	Gu et al. (2016)
In situ removal of by‐product (water) by vacuum	Glycerol + stearic acid	*Novozyme 435*	Residual lipase activity after 144 h	—	92%	Bi et al. (2020)
In situ removal of by‐product (water) by NADES addition (Choline chloride + urea) into the substrates	Glycerol + ω‐3 PUFA	*Novozyme 435*	TAG yield at 50^th^ h	22.1%	49.5%	Xu et al. (2017)
In situ removal of by‐products (medium‐chain fatty acid) by membrane reactor	MCT + DHA/EPA	*Rhizomucor miehei s*upported on macroporous ion exchange resin (*Lipozyme IM*)	Fatty acid incorporation after 80 h	∼25%	∼40%	Xu et al. (2000)

Lipozyme TL IM is immobilized *Thermomyces lanuginosa* supported on silica granulation; Lipozyme RM IM is immobilized *Rhizomucor meihei* supported on microporous anion exchange resin; *Novozyme 435* is immobilized Lipase B from *Candida antarctica* supported on microporous acrylic resin (LEWATIT P OC 1600).

Abbreviations: 4‐HNE, 4‐hydroxynonenal; EDTA, ethylenediamine tetraacetic acid; *k*_d_, deactivation rate constant; MDA, malondialdehyde; PUFA, polyunsaturated fatty acid.

This section reviews current methodologies and novel technologies aimed at improving the operational stability of lipases through strategic substrate management in biocatalytic systems. Emphasis is placed on the interplay between substrate composition and enzyme performance, highlighting both the opportunities and challenges in designing resilient and economically viable enzymatic processes.

### 6.1 Pretreatment With Protein Constituents to Neutralize Reactive Aldehydes

Lipid oxidation generates aldehydes, such as MDA and 4‐HNE, that are known to inactivate lipases through covalent modification of nucleophilic amino acid residues, particularly cysteine thiols. In a study, Pirozzi (2003) investigated a mitigation strategy involving the preincubation of oxidized oil substrates with albumin, a thiol‐rich protein. Albumin effectively scavenges reactive aldehydes, retaining 83.1% and 80.9% of residual lipase activity after 24 h in the presence of 4‐HNE and MDA, respectively. In comparison, samples without pretreatment maintained only 78.6% and 73.5% activity. This protective effect arises because albumin prevents reactive aldehydes from interacting with critical residues such as cysteine, within the lipase‐active site. The study demonstrated that pretreatment with albumin significantly reduced the inactivating effects of both MDA and 4‐HNE, with optimal protective effects observed at concentrations up to 0.2 mM. Beyond this concentration, no further restoration in lipase stability was noted, indicating aldehyde‐induced inactivation extends beyond cysteine modification alone. Other nucleophilic residues, such as lysine and histidine, may also undergo similar modification, highlighting the need for a broader understanding of protein–carbonyl interactions in lipase deactivation (Pirozzi 2003).

Complementary findings were reported by Binder et al. (2006), who employed textured soy proteins as a pretreatment for oxidized lipid substrates. Precontacting oxidized oils with soy protein resulted in more than 2.5‐fold increase in lipase half‐life compared to untreated controls (Binder et al. 2006). This approach offers several advantages for industrial application: soy proteins are food‐grade, widely available, and generally recognized as safe (GRAS), making them suitable for use in food processing.

Collectively, these studies support the application of protein‐based aldehyde scavengers as a viable strategy to enhance lipase stability under oxidative conditions. By sequestering reactive carbonyl species prior to enzymatic processing, such pretreatments help preserve catalytic function and extend operational lifespan of lipase in systems utilizing partially oxidized or lower grade lipid substrates.

Despite these promising outcomes, the industrial implementation of protein‐based mitigation strategies poses several technical and economic challenges. A major concern is the need to remove residual protein postreaction, particularly in food or pharmaceutical applications where purity standards are stringent. This necessitates additional downstream processing steps, increasing operational complexity and cost. High‐purity proteins such as albumin, which is used in a wide range of biomedical applications, are economically impractical for edible oil refining at scale, due to sourcing limitations and high material costs. Protein purification cost‐effectiveness is strongly scale‐dependent, with most processes viable only at scales under 1000 kg per year, orders of magnitude below edible oil refining throughput (Decker et al. 2024). Even with more affordable alternatives such as soy protein, potential drawbacks remain. These include allergenicity concerns, possible interactions with the lipase or substrate, and issues related to product labeling and regulatory compliance. Moreover, integrating protein‐based pretreatment into continuous or semicontinuous production systems is nontrivial. Challenges such as increased viscosity, risk of fouling in fixed‐bed reactors, and pressure drops in flow‐through systems must be addressed through careful process design and engineering.

In summary, while protein‐based scavengers present a chemically elegant and conceptually appealing method for mitigating aldehyde‐induced lipase inactivation, their adoption at industrial scale requires thorough evaluation. Future research should focus on optimizing protein types and concentrations, assessing compatibility with different process configurations, and developing integrated systems that balance enzymatic performance, economic feasibility, and regulatory compliance.

### 6.2 Pretreatment With Adsorbents to Remove Oxidative Degradation Products

A more probable strategy for enhancing lipase stability in biocatalytic system is the pretreatment of lipid substrates using adsorbent materials. These adsorbents function by physically binding and removing unwanted compounds, such as oxidation products, trace metals, pigments, phospholipids, and polar impurities, which are known to contribute to enzyme deactivation. Commonly used in edible oil refining, materials such as bleaching earth (clay‐based) and activated carbons are recognized for their high surface areas and adsorption capacities. While traditionally applied to improve oil clarity, color, and odor, these treatments also reduce the oxidative burden of the substrates, thus providing a protective effect for lipase catalysts during bioprocessing.

In an earlier comparative study, Nezu et al. (1994) evaluated several purification methods for oxidized high‐oleic sunflower oil, including bleaching, neutralization followed by bleaching, and silica‐gel chromatography. When these pretreated oils were used in lipase‐catalyzed reaction, all methods demonstrated varying degrees of improvement in enzyme stability. Notably, silica‐gel chromatography offered the greatest benefit, attributed to its superior removal of secondary oxidation products, as indicated by significantly reduced *p*‐AnV values. Notably, bleaching alone proved ineffective in eliminating trace metal contaminants, such as ferrous ions, underscoring the limitations of single‐step adsorbent treatments in fully preparing lipid substrates for enzymatic applications (Nezu et al. 1994).

Further validation of this approach was provided by Ibrahim et al. (2008), who investigated lipase stability during a 200‐h continuous process using lightly oxidized sunflower and fish oils (PV: 2.6–4.1 mEq/kg). Despite the low‐oxidation levels, lipase activity declined progressively, remained with only 50% residual activity by the end of the experiment. Incorporating an online purification module, consisting of a precolumn packed with various adsorbents, including molecular sieves, activated carbon, and spent *Lipozyme TL IM*, substantially improved performance. The observed deactivation constant (*k*_d_) and half‐life improved by 3.1‐, 7.4‐, and 4.1‐fold, respectively, highlighting the significant protective effect of adsorbent‐based removal of trace oxidative degradation products (Ibrahim et al. 2008).

A similar strategy was employed by Corzo‐Martínez et al. (2018), who applied a mild bleaching step to *Echium* oil using activated clay and carbon prior to enzymatic butanolysis for γ‐linolenic acid (GLA) and stearidonic acid (SDA) enrichment. This pretreatment reduced the PV from 10.6 to 1.0 mEq/kg and the *p*‐AnV value from 4.2 to 3.0, effectively lowering both primary and secondary oxidation product concentrations. As a result, the immobilized lipase attained 905 h half‐life (Corzo‐Martínez et al. 2018).

The effectiveness of an adsorbent depends on multiple physicochemical parameters, including surface polarity, pore size distribution, surface area, and adsorption kinetics. Different oxidation by‐products exhibit varying affinities to these materials: polar degradation products, such as hydroperoxides and aldehydes, are more effectively adsorbed by clay minerals (e.g., zeolites) and silica, while nonpolar or aromatic compounds require carbon‐based adsorbents for effective removal (Liang et al. 2023). Consequently, adsorbent selection must be tailored to the impurity profile of the substrate and specific requirements of the enzymatic process.

Pretreatment with adsorbents presents a practical, scalable, and economically viable approach to enhance lipase stability in oxidative environments. Supporting this, a techno‐economic analysis on sodium hydroxide activated coconut coir priced the bio‐adsorbent at USD95 per metric ton, with a payback period of 2.95 years and an ROI of 39.49% (L. Ifa et al. 2022). By reducing exposure to peroxides, metal ions, and other pro‐oxidants, absorbents can significantly extend enzyme lifespan, improve catalytic consistency, and lower costs associated with enzyme replenishment. These benefits are particularly valuable in continuous or large‐scale biocatalytic systems, where process reliability and cost‐efficiency are paramount. Future work should focus on developing adsorbents with tailored selectivity, either for broad‐spectrum removal or targeted elimination of specific degradation products, to further improve lipase performance in challenging substrate environments.

### 6.3 Pretreatment With Antioxidant to Protect Sensitive Lipid Substrate From Oxidation

Due to their efficacy, ease of application, and cost‐effectiveness, antioxidants have long been utilized to stabilize edible oils and processed foods. Antioxidants functions as effective inhibitors of lipid peroxidation, interrupting the radical chain reactions that initiate and propagate oxidative degradation (Taghvaei and Jafari 2015). More recently, their role is being explored in enzymatic processes involving lipases, where they serve not only to preserve substrate integrity but also to indirectly protect the enzyme from inactivation by oxidation by‐products.

Teixeira et al. (2014) and Vilas Bôas et al. (2021) investigated this concept by incorporating cocoa butter, a natural antioxidant‐rich fat containing β‐sitosterol and various tocopherol isomers (α, β, and γ), into the enzymatic glycerolysis of babassu oil for MAG production. In the continuous flow system operated over 192 h, the MAG yield remained consistently high in both the antioxidant‐supplemented (25.2%) and inert gas‐protected (24.1%) systems, whereas it declined substantially to 18.2% in the unprotected control. These findings clearly demonstrate that antioxidants can confer protection to lipase performance on par with inert gas environment, by stabilizing the oxidative state of the lipid substrate. The authors proposed that cocoa butter could serve as a cost‐effective and food‐compatible alternative to inert gas system, particularly in large‐scale or continuous biocatalytic systems where inert gas infrastructure may be prohibitively expensive or logically complex (Teixeira et al. 2014; Vilas Bôas et al. 2021).

Tert‐butylhydroquinone (tBHQ) is recognized for its potent antioxidative efficacy within lipid matrix. Binder et al. (2006) implemented several mitigation strategies, including the incorporation of a precolumn packed with texturized soy protein, the addition of tBHQ to the substrate oil, and the operation of the biosynthesis under inert nitrogen atmosphere. The combined approaches markedly enhanced the enzyme's stability, extending its half‐life to 42 days compared to only 6 days under the control conditions (Binder et al. 2006). In their study on enzymatic acidolysis, Xu et al. (2005) reported the inclusion of antioxidants tocopherols and ethylenediamine tetraacetic acid (EDTA) showed slightly better incorporation of fatty acids into oil than control experiments after 24 h. However, the group cautioned that over extended operation, residual antioxidants and accumulated oxidation by‐products might alter the microenvironment of the immobilized enzyme, potentially affect enzyme conformation, promote fouling, or reduce catalytic turnover, particularly in PBR or fixed‐bed reactor systems (Xu et al. 2005). These long‐term effects highlight the need for deeper understanding of antioxidant–enzyme interactions within immobilized systems, especially under continuous flow conditions.

Collectively, these studies suggest that antioxidant incorporation can be an effective strategy to preserve lipase functionality during oxidative stress. However, the success of this approach depends on several factors, including antioxidant stability over prolonged operation, compatibility with immobilization matrices and support materials, potential accumulation of antioxidants within the reactor, and impact on substrate–enzyme interfacial dynamics and mass transfer efficiency. While antioxidant pretreatment offers a chemically straightforward and scalable route to enhancing enzymatic performance, it must be carefully optimized for specific process configurations. Future research should focus on understanding the mechanistic interactions between antioxidants, enzymes, and substrate phases, particularly under industrially relevant conditions. Additionally, screening for food‐safe, nontoxic, and cost‐effective natural antioxidants with minimal downstream processing requirements will be critical to ensuring broad applicability in commercial enzymatic lipid processing.

### 6.4 Process Design Strategies

#### 6.4.1 Reactor Design

While batch stirred‐tank reactors (STRs) are commonly employed in enzymatic lipid synthesis, they often exhibit lower operational stability than continuous PBRs. This difference primarily arises from the greater mechanical sheer stress imposed on immobilized enzymes in STRs and the larger fluctuations in substrate‐to‐product ratios within the reaction medium. Continuous PBRs promote constant removal of products and substrates not consumed by the reaction, reducing the potential inhibition of enzyme activity. For example, Gu et al. (2016) reported a markedly longer lipase half‐life in a continuous PBR (140 h) compared with a batch STR (24 h) during the synthesis of SL dioleoyl palmitoyl‐rich TAGs (Gu et al. 2016). Notably, substrate quality can become a more critical factor in continuous systems. Prolonged enzyme exposure, even to low concentrations of oxidized lipid compounds, can lead to gradual inactivation. In contrast, in batch reactors, enzyme deactivation was observed only under highly oxidized conditions, at PV and TBARS levels rarely encountered in commercial oils (Pirozzi 2003).

Additionally, process yield and lipase stability may be compromised in continuous systems due to poor phase compatibility between hydrophilic and hydrophobic substrates and deactivation effect of certain substrates toward enzyme, for instance, between glycerol and babassu oil, during enzymatic lipid processing (Teixeira et al. 2014). Under such conditions, fed‐batch STR can serve as a practical intermediate approach. In fed‐batch operation, substrates are generally introduced to maintain subinhibitory concentrations while the reaction volume increases over time, thereby extending enzyme longevity and improving overall process stability.

An alternative continuous design that addresses these mechanical and compatibility challenges is the FBR. By employing upward fluid flow to suspend the immobilized enzyme particles, the FBR eliminates the high compaction and localized shear stress typical of PBR while maintaining continuous operation. Osorio et al. (2005) used *Novozyme 435* in a FBR with palm stearin and soybean oil, achieving a half‐life of 408 h, substantially longer than any PBR study. The FBR's gentle fluidization minimizes particle–particle friction and reduces localized heating, which together limit the formation of oxidation products, thereby preserving enzyme activity over extended periods.

#### 6.4.2 Reactor Configuration

The choice of reactor configuration directly influences the extent to which oxidized compounds and impurities interact with lipase. Strategic reactor design can localize or limit the enzyme's exposure to these inhibitory species, thereby improving operational stability.

Multistage PBRs offers a compartmentalized setup that buffers the enzyme from oxidative stress. Won et al. (2012) investigated the enzymatic interesterification of fully hydrogenated canola oil and soybean oil using single‐, double‐, and triple‐stage PBRs. They observed a clear enhancement in lipase stability, with enzyme half‐life increasing from 122 h in a single‐stage system to 198 h in a triple‐stage setup. This improvement was attributed to the “sacrificial” function of the first stage, where oxidized components and other impurities were adsorbed or neutralized. Replacing the enzyme bed in the first stage restored system performance, reinforcing the protective benefit of staged configurations (Won et al. 2012). Similar finding was obtained by Cozentino et al. (2022) that serial‐double PBRs gave rise to longer lipase half‐life than single‐stage PBR (Cozentino et al. 2022).

#### 6.4.3 In Situ Removal of Inhibitory Compounds

##### 6.4.3.1 Strategies for Water Removal

In esterification reactions, water produced as a by‐product can accumulate around the enzyme support, promoting hydrolysis and resulting in partial loss of the enzyme's native conformation due to disruption of structural interactions. Bi et al. (2020) demonstrated that applying vacuum during such reactions helped remove water continuously, preserving 92% of initial catalytic activity over 12 cycles, at 100°C, a temperature higher than the common tolerable range that enzyme can withstand (Bi et al. 2020). A reduced water content around protein molecules modifies the hydrogen bond interactions between water and protein, thereby increasing the denaturation temperature. In addition, the application of vacuum provided a relatively inert atmosphere which may enhance the thermostability of enzymes.

As an alternative approach, hydrophilic natural deep eutectic solvents (NADES) have been employed to actively sequester water from the reaction medium. For instance, a DES composed of choline chloride and urea (1:2, mol/mol) demonstrated high water absorbency in esterification synthesis of n‐3 PUFA‐enriched TAG, effectively shifting the reaction equilibrium toward product formation (Xu et al. 2017). Unlike vacuum‐assisted removal, which continuously strips water vapor from the headspace, NADES acts as an in situ water scavenger through hydrogen bonding interactions, offering a mild, nonthermal means to maintain a low‐water environment. Following the reaction, the hydrophilic NADES can be readily separated from the reaction products by simple physical methods, enabling solvent recovery and reuse.

##### 6.4.3.2 Membrane‐Integrated Systems for Inhibitory Compound Management

Membrane reactors offer selective removal of undesirable compounds from the reaction medium, maintaining a more stable environment for the enzyme. Xu et al. (2000) demonstrated the use of a membrane bioreactor in the synthesis of SLs from MCTs and EPA/DHA‐rich oils. The membrane selectively removed short‐chain fatty acids (C8:0/C10:0), which could otherwise accumulate and negatively affect enzyme performance. This approach led to a 15% improvement in DHA/EPA incorporation and maintained enzyme stability for over 80 h, compared to control systems that experienced early performance decline (Xu et al. 2000).

## 7 Industrial Challenges

Despite advances in lipase‐catalyzed SLs synthesis, translating laboratory successes into economically viable processes remains difficult. A key issue is that scale‐up investment decisions are often based on highly optimized systems using standard, well‐defined substrates. This approach overlooks the inherent variability of real‐world feedstocks, such as agro‐industrial by‐products or oxidatively compromised lipids. The resulting misalignment between laboratory conditions and industrial reality leads to overestimated process robustness and jeopardizes return on investment.

Further complicating this matter the limited understanding of immobilized lipase tolerance to substrate variability. Enzyme performance is sensitive to oxidation products, FFA compositions, and water activity. However, systematic evaluations of enzyme resilience under such nonideal and dynamic conditions in various reactor designs remain scarce. As a result, industrial processes are often designed with overly idealistic operational boundaries, leaving them vulnerable to feedstock deviations. Sudden activity declines, accelerated deactivation, and inconsistent product quality are addressed reactively, leading to increased downtime and reduced reliability.

## 8 Future Research Directions

Future research must shift from idealized optimization to robustness‐driven process design. Systematic studies should map tolerance limits of immobilized lipases across variable, low‐grade feedstocks relevant to food by‐product valorization. Standardized protocols for assessing enzyme stability under realistic conditions are needed. A practical proposal is a low‐cost “stress‐test” panel of challenge substrates to benchmark enzyme robustness before scale‐up. Real‐time monitoring tools and data‐driven models should be integrated to predict performance and detect early deactivation. Adaptive control strategies should be concrete, such as feeding rate modulation or in‐line scavenger addition triggered by peroxide or water activity sensors to reduce inhibitory by‐products before they cause irreversible enzyme inactivation.

Mechanistic studies of enzyme–substrate interactions under heterogeneous conditions are essential, as is designing more resilient biocatalysts. Critically, future research should ask: *“What substrate quality is ‘good enough’?”* rather than assuming high purity is always better.

Collectively, these efforts will establish a predictive framework for biocatalytic process design, reducing scale‐up risks and ensuring that industrial implementation aligns with technical feasibility and economic reality, essential for the food industry, where feedstock variability is the rule, not the exception.

## 9 Conclusions

Synthesis of SL involves enzymatic restructuring of TAGs to achieve a specific fatty acid composition and positional distribution. However, maintaining and optimizing lipase operational stability requires deliberate control of substrate characteristics (oxidative status, water activity, and FFA composition), particularly vital when utilizing PUFAs and agro‐industrial by‐products. Ideally, upstream lipid pretreatment, targeted antioxidant strategies, and advanced reactor designs are essential for scalable production of high‐value SLs, to support functional food and nutraceutical applications. However, from an industrial standpoint, a persistent challenge remains: scale‐up decisions based on idealized substrates overlook real‐world feedstock variability, leading to overestimated process robustness and jeopardizes return on investment. Future research must shift to robustness‐driven design. Priorities include: (i) stress‐test panels using spiked or aged lipids; (ii) real‐time adaptive control (e.g., sensor‐triggered feeding modulation); and (iii) defining “good enough” substrate quality prior to pilot‐scale injection, with a proposed baseline of PV ≤ 5 mEq/kg and AnV ≤ 5, generally aligns with industrial freshness standards for refined oils. Collectively, these substrate‐centered strategies and a robustness‐driven research agenda establish a predictive, economically grounded framework for scalable biocatalytic processes, essential for the food industry, where feedstock variability is the rule, not the exceptions Supporting Information.

## Author Contributions

**Evelyn Ling Lee**: conceptualization, investigation, data curation, visualization, writing – original draft. **Eng‐Seng Chan**: conceptualization, writing – review and editing. **Lee Fong Siow**: supervision, writing – review and editing. **Cher Pin Song**: writing – review and editing. **Yee‐Ying Lee**: supervision, writing – review and editing, conceptualization, resources.

## Declaration of Generative AI and AI‐Assisted Technologies in the Writing Process

Artificial intelligence‐assisted tools were used to support language editing (improving grammar, coherence, and flow) and the preparation of conceptual mechanistic illustrations. The authors reviewed, edited, and verified all outputs, and take full responsibility for the scientific content and accuracy of the manuscript.

## Funding

This research work is supported by the School of Science, Monash University Malaysia.

## Conflicts of Interest

The authors declare no conflicts of interest.

## Supporting information




**Supporting Information**: sup‐0001‐SuppMat‐jfds71175.docx

## Data Availability

Data will be made available upon request.
