# Supplementary material for: Lipase Stability in Structured Lipid Synthesis: The Interplay of Substrate Characteristics and Strategies to Improve Its Operational Performance—A Critical Review
Source: J Food Sci. 2026 Jun 24;91(6):e71175. doi: 10.1111/1750-3841.71175 (PMC13292194; doi:10.1111/1750-3841.71175)
Supplement: Supplementary file 1 — Supporting Information: sup‐0001‐SuppMat‐jfds71175.docx [file JFDS-91-0-s001.docx]

**List of Abbreviations**

| **Abbreviation** | **Definition** |
| --- | --- |
| 4-HHE | 4-hydroxy-2-hexenal |
| 4-HNE | 4-hydroxy-2-nonenal |
| ALA | Alpha linolenic acid |
| ARA  *C. parapsilosis* | Arachidonic acid  *Candida parapsilosis* |
| DAGs | Diacylglycerol |
| DHA | Docosahexaenoic acid |
| DPA | Docosapentaenoic acid |
| EDTA | Ethylenediamine tetraacetic acid |
| EPA | Eicosapentaenoic acid |
| FAEEs | Fatty acid ethyl esters |
| FAMEs | Fatty acid methyl esters |
| FBR | Fluidized bed reactor |
| FFA | Free fatty acids |
| GLA | γ-linolenic acid |
| GMP | Good manufacturing practices |
| HMFS | Human milk fat substitutes |
| *k_d_* | Deactivation constant |
| MAGs | Monoacylglycerols |
| mEq/kg | milliequivalents of active oxygen per kilogram of fat |
| MDA | Malondialdehyde |
| MLCTs  NADES | Medium- and long-chain triacylglycerols  Natural deep eutectic solvents |
| *p*-AnV | *para*-anisidine values |
| pHMB | p-hydroxymercuribenzoate |
| PBR | Packed bed reactor |
| PUFAs | Polyunsaturated fatty acids |
| PV  *R. meihei*  *R. oryzae* | Peroxide value  *Rhizomucor meihei*  *Rhizopus oryzae* |
| SDA | Stearidonic acids |
| SLs | Structured lipids |
| STRs | Stirred-tank reactors |
| TAGs | Triacylglycerols |
| TBARS | Thiobarbituric acid reactive substances |
| tBHQ | Tert-Butylhydroquinone |
